# Clinical Implications of Inflammation in Acute Myeloid Leukemia

**DOI:** 10.3389/fonc.2021.623952

**Published:** 2021-02-22

**Authors:** Christian Récher

**Affiliations:** Service d’Hématologie, Centre Hospitalier Universitaire de Toulouse, Institut Universitaire du Cancer de Toulouse Oncopole, Université Toulouse III Paul Sabatier, Centre de Recherches en Cancérologie de Toulouse, Toulouse, France

**Keywords:** acute myeloid leukemia, inflammation, glucocorticoids, dexamethasone, RUNX1, FLT3, leukemic stem cells, chemoresistance

## Abstract

Recent advances in the description of the tumor microenvironment of acute myeloid leukemia, including the comprehensive analysis of the leukemic stem cell niche and clonal evolution, indicate that inflammation may play a major role in many aspects of acute myeloid leukemia (AML) such as disease progression, chemoresistance, and myelosuppression. Studies on the mechanisms of resistance to chemotherapy or tyrosine kinase inhibitors along with high-throughput drug screening have underpinned the potential role of glucocorticoids in this disease classically described as steroid-resistant in contrast to acute lymphoblastic leukemia. Moreover, some mutated oncogenes such as *RUNX1*, *NPM1*, or *SRSF2* transcriptionally modulate cell state in a manner that primes leukemic cells for glucocorticoid sensitivity. In clinical practice, inflammatory markers such as serum ferritin or IL-6 have a strong prognostic impact and may directly affect disease progression, whereas interesting preliminary data suggested that dexamethasone may improve the outcome for AML patients with a high white blood cell count, which paves the way to develop prospective clinical trials that evaluate the role of glucocorticoids in AML.

## Introduction

Acute myeloid leukemia (AML) is a myeloid malignancy induced by the oncogenic transformation of hematopoietic progenitors in the bone marrow leading to the destruction of blood tissue responsible for acute pancytopenia, severe bleeding, and infection (1). For over 40 years, intensive treatment of AML has been based on a combination of cytarabine and an anthracycline as induction chemotherapy, followed by intermediate to high-dose cytarabine consolidation and possibly allogeneic stem cell transplantation aimed at curing the disease. On the other hand, older patients or those deemed unfit for high intensity treatment received low-dose cytarabine or more recently, hypomethylating agents, both of which induce few complete responses and no hope for cure (2). While it used to be commonly acknowledged that there was no progress in the treatment of AML compared to other hematological malignancies, recently there has been a major effort to understand the disease and to develop novel promising drugs that specifically target recurrent gene mutations, apoptotic pathways, and cell surface antigens or by reformulating classical cytotoxic agents. Midostaurin, gemtuzumab ozogamycin, glasdegib, venetoclax, ivosidenib, enasidenib, gilteritinib and CPX-351 were approved by the Food and Drug Administration for AML patients in less than 2 years between 2017 and 2018, which brings hope for this highly fatal disease and many opportunities for clinical research (3, 4).

In the era of next-generation sequencing, considerable progress has been made in understanding leukemogenesis, the genetic diversity of AML, gene-gene interactions, clonal evolution, and the assessment of treatment responses (5–11). Several functional categories of recurrent gene mutations that affect transcription factors, cell signalization, nucleophosmin, epigenetics, DNA methylation, and RNA splicing or the cohesin complex interact to produce the main hallmarks of cancer and transform hematopoietic progenitors into AML cells. While much attention has been focused on these molecular alterations in recent years, another cancer hallmark, tumor-promoting inflammation, has on the contrary, received little attention in the field of AML (12). However, clinical findings, previous data as well as recent advances in the description of the tumor microenvironment of AML, including a comprehensive analysis of the leukemic stem cell niche, indicate that inflammation may play a role in many aspects of AML such as disease progression, chemoresistance, and myelosuppression or leukostasis syndrome (13). In this review, we highlight emerging data on the clinical and preclinical impact of inflammation in AML and the first attempts to modulate this phenomenon with anti-inflammatory drugs such as dexamethasone.

## Clinical Practice

### The Inflammatory Response in AML Patients Treated With Intensive Chemotherapy

In routine clinical practice it is not uncommon to observe fever induced by chemotherapy in AML patients (14). The so-called cytarabine syndrome, which includes fever, myalgia, bone pain, maculopapular rash, conjunctivitis, malaise, and occasionally pericarditis, has long been described (15). Steroids can help to prevent or treat this syndrome. Fever is induced by endogenous pyrogens such as the interleukins IL1-α, IL-1β, IL-6, and TNF-α involved in the inflammatory response. It has been shown that high-dose cytarabine treatment induces a release of TNF-alpha followed by the sequential release of other proinflammatory cytokines (16, 17). This is consistent with the findings in a series of unselected AML patients treated by intensive induction chemotherapy with 3–5 days of anthracyclines and 7 days of cytarabine where inflammatory markers including serum ferritin and CRP levels were significantly increased at day 8 (as much as a 3.5-fold increase compared to baseline) (18). These data indicate that intensive chemotherapy, whether with 3 + 7 induction or high-dose cytarabine consolidation, often induces an inflammatory response in AML patients. The physiopathological consequences of this response remain unclear. In this context, inflammation may be further aggravated by infection during chemotherapy-induced myelosuppression or hyperinflammatory states such as hyperferritinemic syndrome or hemophagocytic lymphohistiocytosis, an immune dysregulation characterized by severe organ damage induced by an exacerbated inflammatory response and uncontrolled T-cell and macrophage activation. Secondary hemophagocytic lymphohistiocytosis typically occurs in association with severe infections or malignancies (19, 20). In a large series of AML patients treated by intensive chemotherapy, ~10% of the patients were noted to have fever, very high serum ferritin levels and bone marrow hemophagocytosis accompanied by hepatomegaly, pulmonary or neurological symptoms, liver abnormalities, a lower platelet count, higher levels of C-reactive protein and prolonged pancytopenia. The possibility of an infectious etiology that functions as a trigger for hyperinflammation, including bacterial, fungal, or *Herpesviridae* infections, was documented in 75% of the cases. In this study, hemophagocytic lymphohistiocytosis was associated with poor overall survival, which suggests that inflammation may impact prognosis in AML patients (21). With the current SARS-CoV-2 pandemic, hyperferritinemic syndromes and the pathogenic role of ferritin in critically ill patients are receiving considerable attention in terms of prognosis, clinical management, and therapeutic intervention (22–24).

### Serum Ferritin in AML: More Than a Prognostic Marker

The role of cytokines and inflammatory pathways in AML was recently reviewed in other studies (13, 25, 26). Special attention was paid to IL-1 and IL-6 which have been associated with a poor prognosis, chemoresistance, and myelosuppression in AML (27–30). Here, we focus on recent data regarding the impact of ferritin in AML. In fact, independently of hyperinflammatory syndromes, it has also been observed that most AML patients have elevated serum ferritin levels at diagnosis, even younger patients with *de novo* AML who in general have had no red blood cell transfusion at the time of diagnosis (18, 31). This suggests that the increase in ferritin is most likely due to an underlying inflammatory condition rather than iron overload or liver damage. A recent study of a cohort of 525 AML patients treated with intensive chemotherapy showed that a higher ferritin level was significantly associated with age, higher CRP levels, leukocytosis, FAB M4/M5 subtypes, *NPM1*, and *FLT3*-ITD mutations (18). More importantly, serum ferritin was a risk factor for a poor response to treatment, early death, the incidence of relapse and survival endpoints independently of cytogenetics, molecular alterations or allogeneic stem cell transplantation. The median OS was 41.0 months in patients with serum ferritin ≤900 µg/l (3-fold the UNL) compared to 14.4 months in patients with >900 µg/l. Other studies have reported a poor prognostic impact of ferritin in AML patients (31–33). It is also noteworthy that in most cases CRP levels are usually elevated at diagnosis, but CRP had no prognostic impact in patients treated with intensive chemotherapy when serum ferritin was in a multivariate model (31, 33).

The fact that there is a significant association between serum ferritin levels and a poorer response to induction chemotherapy as well as a higher incidence of relapse would suggest that in addition to this statistical correlation ferritin also plays a role in chemoresistance. Overexpression of H-ferritin contributes to lymphomagenesis and has been involved in resistance to chemotherapy agents, including doxorubicin, which induces oxidative stress (34–36). In AML, ferritin protein expression in patient samples was correlated with a response to cytarabine *in vitro* (18). Furthermore, high ferritin levels before allogeneic stem cell transplantation have been associated with a higher risk of relapse and lower overall survival (37). With regards to the risk of relapse, this association remains unexplained and is not necessarily related to iron overload in patients who are transfused during induction and consolidation. Rather, these recent data could indicate that pre-transplantation ferritin levels are a surrogate marker of residual disease before transplantation.

If ferritin does play a role in disease progression and treatment resistance, what are the potential mechanisms for this? Ferritin is a 450 kDa protein that consists of 24 subunits of H-ferritin and L-ferritin encoded by the *FTH1* (ferritin heavy chain 1) and *FTL* (ferritin light chain) genes (38). Ferritin is a multi-functional protein that regulates several biological processes at both extra and intracellular levels that could be relevant in AML biology including cell proliferation, immunosuppression, angiogenesis, and chemoresistance (39). Inflammatory cytokines including IL-1β, IL-6, TNF-α, and growth factors such as insulin-like growth factor (IGF-1), which are responsible for ferritin expression through NF-kB activation, are frequently overexpressed in AML and play a crucial role in the leukemic stem cell niche (13, 29, 40, 41). In addition, extracellular ferritin could also act as a pro-inflammatory cytokine and induces NF-kB signaling (42). Therefore, ferritin synthesis induced by inflammatory cytokines could also play a role in a positive feedback loop that sustains the activation of nuclear factor-κappa B (NF-kB) in AML cells which is critical for chemoresistance and leukemic stem cell survival (43, 44). AML transcriptomic databases reveal that *FTH1* and *FTL* are frequently overexpressed regardless of genetic subgroup in both AML bulk and leukemic stem cells compared to normal hematopoietic stem cells. In this context, it has been shown that glioblastoma cancer stem cells are epigenetically programed to scavenge iron more effectively than other tumor cells and require transferrin receptor and ferritin to propagate tumors (45). Ferritin is a growth factor for AML cells and its antioxidant activity may decrease the efficacy of cytotoxic agents such as anthracyclines (34, 35, 46). Moreover, H-ferritin induced by TGF-b can exert a suppressive effect on normal myelopoiesis (47). Overall, these data suggest that ferritin may impact leukemic stem cell biology within an inflammatory niche. Intracellular H-ferritin could protect AML cells from chemotherapy by inducing anti-apoptotic and anti-oxidative response while extra-cellular H-ferritin may contribute to local inflammation and alter normal bone marrow hematopoietic progenitors contributing to myelosuppression related to disease evolution and chemotherapy (48, 49).

Another point for discussion is the cellular origin of ferritin in AML, although it is thought to reside in the mononuclear phagocyte system and in hepatocytes in non-malignant conditions (50–52). Macrophages activated by an aberrant inflammatory response are likely to be the main source of serum ferritin overproduction in AML patients (50). However, a significant association was found between ferritin levels and hyperleukocytosis (tumor burden). AML cells express higher intracellular ferritin protein levels than normal peripheral blood mononuclear cells. In a previous study, leukemic cells from patients with acute monocytic leukemia (AML FAB M5) showed the highest ferritin levels (53). Furthermore, ferritin was detected in the supernatant of AML cell lines incubated in serum free media for 24 h, which suggests that AML cells may release ferritin in extra-cellular media. In addition, anthracyclines such as doxorubicin and daunorubicin may directly interact with the iron response element hairpin loops in the 5′-UTR of ferritin H- and L-chain mRNAs which could be a direct link between drug exposure and ferritin production (54). Doxorubicin has been shown to produce a significant increase in the plasma concentration of transferrin, ferritin, and iron in experimental models (55). Therefore, leukemic cells could contribute to serum ferritin production in AML patients. This hypothesis has also been suggested in previous studies (56, 57).

**Figure 1:**
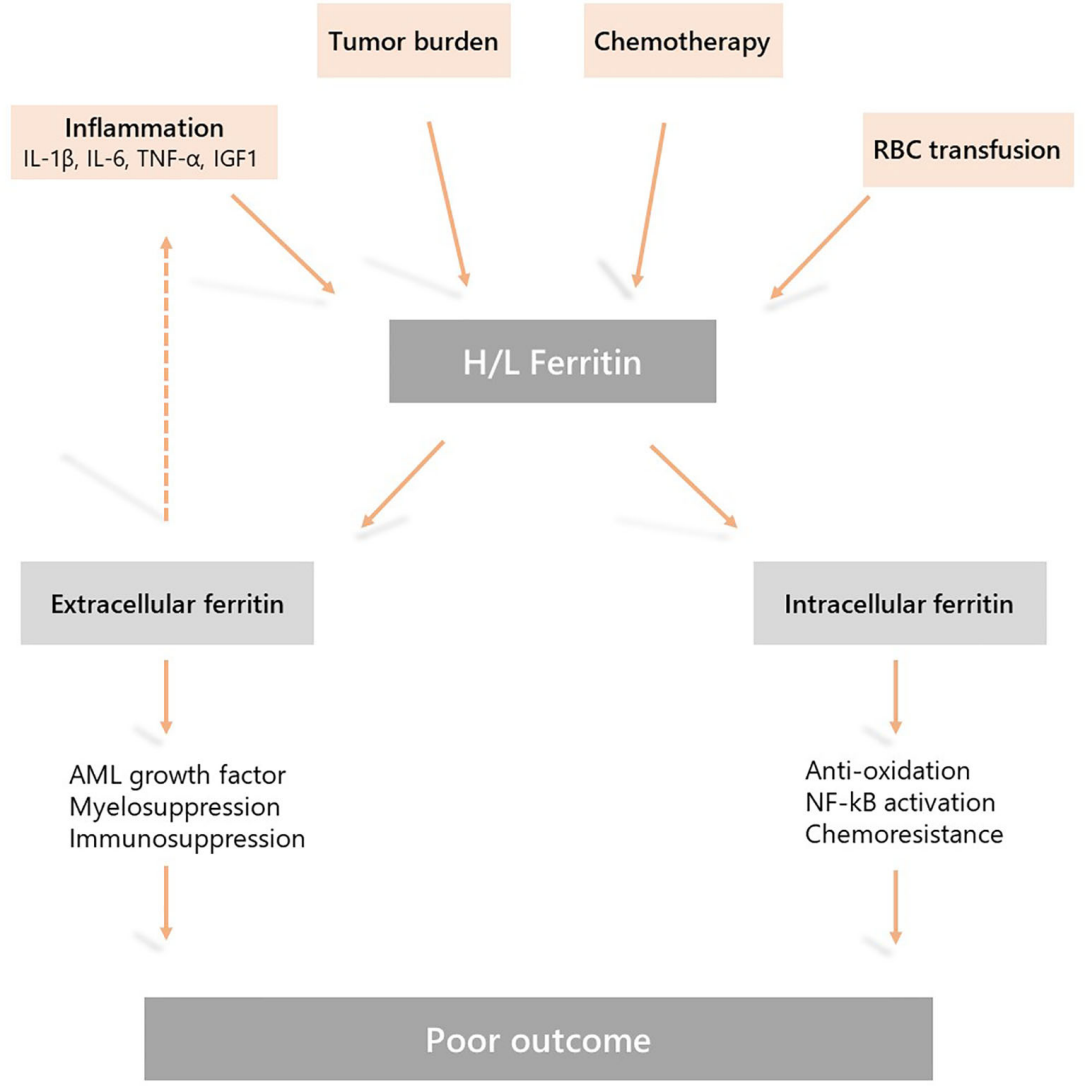
Mechanisms underlying hyperferritinemia in acute myeloid leukemia (AML) and the role of ferritin in AML physiopathology. Elevated serum ferritin levels are due to a dysregulated inflammatory response, chemotherapy, disease burden, and the release by leukemic cells. This is further sustained by iron overload due to red blood cell transfusion during induction and consolidation treatment. Extracellular and intracellular activities of ferritin may induce resistance to chemotherapy, myelosuppression and disease progression resulting in a poor outcome.

Accumulating evidence suggest that in AML increased serum ferritin levels are due to both a dysregulated inflammatory response and disease burden, and may have a direct impact on disease progression and response to therapy. Therefore, pharmacological modulation of this pathway could be a new therapeutic target in AML (**Figure 1**).

### Dexamethasone in Hyperleukocytic AML

Glucocorticoids such as dexamethasone are anti-inflammatory drugs widely used in acute lymphoblastic leukemia (ALL) and other lymphoid malignancies (58). There are only some clinical data that indicate potential action of glucocorticoids in AML. Turkish investigators previously reported their long-term experience with high-dose methylprednisolone in pediatric AML (59). Based on preclinical data showing that methylprednisolone induces AML cell differentiation and apoptosis while stimulating normal myelopoiesis, and on clinical observations of the remarkable antileukemic effect of high-dose methylprednisolone, they investigated the activity of short-term high-dose methylprednisolone treatment (20–30 mg/kg/day not exceeding 1 g/day) in childhood AML (60, 61). They were able to show that methylprednisolone when used according to this schedule as a single agent did induce leukemic cell differentiation and apoptosis in patients. Furthermore, methylprednisolone as pretreatment before high-dose chemotherapy reduced the duration and severity of neutropenia (62). However, no prospective randomized clinical trials were subsequently conducted to confirm these promising data and to generally establish glucocorticoids in the treatment of childhood AML.

In adult AML, dexamethasone has long been used to prevent or treat a severe inflammatory condition known as differentiation syndrome in patients with acute promyelocytic leukemia (APL) treated with all trans-retinoic acid (ATRA) and/or arsenic trioxide, or more recently in AML patients treated with IDH or FLT3 inhibitors (63, 64). In fact, in APL, dexamethasone (10 mg/12 h for at least 3 days) was systematically added to “3+7” + ATRA induction chemotherapy in prevention of the differentiation syndrome in all patients with a white blood cell count (WBC) >10 G/L. Therefore, there is an established clinical experience of the use of glucocorticoids in the context of chemotherapy-induced myelosuppression in AML patients and no harmful adverse events, especially with regard to fungal invasive infections, were reported (63, 65, 66).

**Figure 2:**
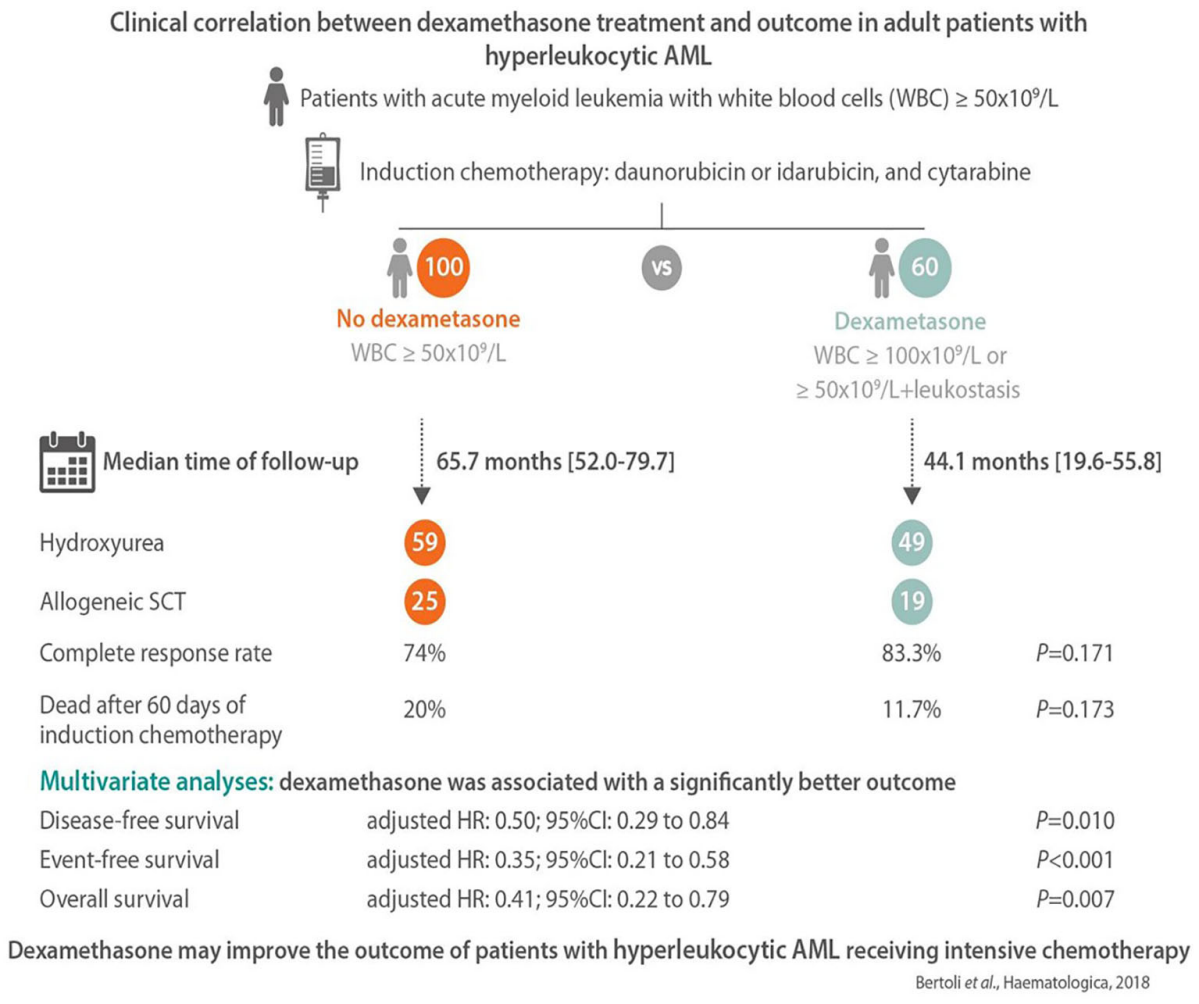
The main results of the DEXAML-00 retrospective study in hyperleukocytic acute myeloid leukemia (AML) patients. Graphical abstract by Haematologica showing the endpoints of a retrospective, monocentric study comparing the addition of dexamethasone to standard induction chemotherapy in AML patients with hyperleukocytosis defined as a white blood cell count ≥50 × 10^9^/L. From 2010 to 2015, 60 patients received dexamethasone (10 mg/12 h, days 1–3) and were compared to 100 patients who did not received dexamethasone from 2004 to 2009. In multivariate analysis, dexamethasone was associated with a significant improvement of disease-free, event-free and overall survival.

Approximately 20% of AML patients present with a high WBC count (>50 G/L) at diagnosis (67). This is a high-risk situation in which the probability of severe complications and early death is increased because of leukemic organ infiltration, severe hemorrhage, tumor lysis syndrome, or disseminated intravascular coagulopathy. Hyperleukocytosis is also associated with leukostasis syndrome in the lung or brain, which can lead to acute respiratory distress syndrome or stroke. Leukostasis induces endothelial injury and activation *via* microvascular invasion, hyperviscosity, leukocytic microthrombi, and oxygen consumption. Mediators of inflammation induced by leukemic blasts and endothelial cells play a central role in the pathogenesis of leukostasis (68). Studies on the molecular mechanisms of leukostasis and leukemic cell invasion have shown that leukemic blasts use integrins and selectins to attach to cytokine-activated endothelium and directly activate endothelial cells by secreting inflammatory cytokines such as TNF-a, IL-1β, and IL-6, which induce the conditions necessary for their adhesion to vascular endothelium, migration to tissues, proliferation, and chemoresistance (68, 69). Because glucocorticoids exert a potent inhibitory effect on cytokine production, dexamethasone has been suggested for use in patients with AML FAB M5 and acute lung injury or acute respiratory distress syndrome who are admitted to the intensive care unit. Compared to historical controls, dexamethasone-treated patients had a significantly lower mortality rate (66). A subsequent study in adult patients admitted to the intensive care unit with respiratory events at the earliest phase of AML also showed that dexamethasone therapy was a factor independently associated with lower mortality on day 28 in the multivariate analysis (70). Moreover, a recent retrospective study that compared hyperleukocytic patients treated with intensive chemotherapy with or without a short course of dexamethasone (DEXAML-00) showed that routine addition of dexamethasone to induction chemotherapy was associated with a significant improvement in the main survival endpoints. In fact, a multivariate analysis showed that dexamethasone was significantly and independently associated with a lower incidence of relapse as well as an improvement in disease-free survival, event-free survival, and overall survival (**Figure 2**) (71).

## Bench Research

### The Mechanism of Action of Glucocorticoids

Glucocorticoid drugs act through different targets and mechanisms to control inflammation (72). After crossing the cell membrane, glucocorticoids bind to the cytoplasmic glucocorticoid receptor (GR) and induce dissociation of molecular chaperones, including heat shock proteins and immunophilins, from the GR (73). The ligand-bound GR displays non genomic cytoplasmic activities that interfere with cell signal transduction pathways such as PI3-kinases, MAP-kinases as well as T-cell receptor signaling. Of course, the main mechanisms of action of GR are in the nucleus (74). First, as a homodimer, ligand-bound GR binds to DNA and acts as a transcription factor inducing the expression of anti-inflammatory response genes. Second, as a monomer, GR may physically interact with transcription factors such as AP-1 and NF-kB, thereby inhibiting their pro-inflammatory activities (75).

### Challenges in Using Glucocorticoids in AML

Unlike ALL, AML blasts are not usually sensitive to pharmacologic doses of glucocorticoids (76). In vitro, it has been shown that glucocorticoids induced cell proliferation in some pediatric AML samples (77). Moreover, a recent study focusing on both BCR-ABL positive ALL and chronic myelocytic leukemia cells demonstrated that strong differences in energetic metabolism driven by B-cell lineage transcription factors explain the difference in glucocorticoids sensitivity between lymphoid and myeloid cells (78). The B-lymphoid transcription factors, PAX5 and IKZF1, which are crucial for early B cell development and repressed in myeloid cells, induce a transcriptional program leading to a state of chronic energy deprivation by restricting glucose uptake. This establishes a metabolic barrier to the leukemogenesis of ALL. This transcriptional program is also associated with up regulation of glucose uptake inhibitors expression including NR3C1 thereby contributing to ALL sensitivity to glucocorticoids. Moreover, mechanisms of resistance to glucocorticoids that has been extensively studdied in ALL may also occur in AML cells including alterations in uptake and efflux by multidrug transporters, GR expression or in downstream apoptotic response (79, 80). Indeed, the regulation of expression of BCL2 family proteins such as the proapoptotic BIM and the anti-apoptotic BCL2 or MCL1 is key to determine the level of glucocorticoid sensitivity (81).

In clinic, glucocorticoids may increase the risk of bacterial or invasive fungal infections by worsening the immunosuppression of AML patients during intensive chemotherapy. Therefore, these drugs have received little attention in the field. However, we discuss below recent emerging preclinical data suggesting that glucocorticoids may have a positive therapeutic impact in certain settings in AML.

### The Action of Glucocorticoids in Chemoresistance

The unexpected impact of dexamethasone observed on the cumulative incidence of relapse suggests a potential antileukemic activity on chemoresistant AML cells (71). In a recent preclinical study, AML cell lines were rendered resistant to cytarabine through chronic exposure to increasing drug concentrations then subjected to genomic and transcriptomic profiling as well as high-throughput testing with 250 clinical oncology compounds (82). It was shown that the acquisition of cytarabine resistance was associated with increased sensitivity to glucocorticoids. Similarly, paired samples from AML patients, while unresponsive at diagnosis, became sensitive to glucocorticoids including dexamethasone, methylprednisolone, and prednisolone at relapse after exposure to a cytarabine-based chemotherapy regimen. In this study, glucocorticoid activity was mainly observed in wild type FLT3 samples whereas mutated FLT3-ITD samples appeared resistant. Resistance to cytarabine was associated with deletion of the *DCK* gene that encodes deoxycytidine kinase, the rate-limiting enzyme in the metabolic activation of cytarabine. Moreover, upregulation of GR protein expression was also observed in cytarabine-resistant AML cells and contribute to the acquisition of glucocorticoid sensitivity. However, it should be noted that in a rat model of AML, dexamethasone decreased the activity of deoxycytidine kinase (83). Meanwhile, in a similar study, the response of 2 DCK-defective murine AML cell lines to 446 FDA approved drugs compared to their cytarabine sensitive parental lines was examined. Once again, cytarabine-resistant cells that lacked functional deoxycytidine kinase were sensitive to prednisolone and dexamethasone in a GR-dependent manner (84). Overall, these studies showed that mechanisms that lead to cytarabine resistance may be linked to the acquisition of dexamethasone sensitivity.

Chemoresistance is also mediated by interactions between AML cells and their microenvironment (85–87). It has been recently demonstrated that an inflammatory and immune interferon-γ signature is associated with chemoresistance (88). In liquid culture, short term dexamethasone treatment with or without cytarabine or doxorubicin showed no synergy or additive effect in genetically diverse AML cell lines. However, in a co-culture system, one week of dexamethasone exposure significantly enhanced cytarabine activity in most AML cell lines, which indicates that glucocorticoids may interfere with soluble factors or cellular interactions involved in microenvironment-induced resistance (71, 89). Furthermore, using a patient-derived xenograft (PDX) model of cytarabine resistance, it was shown that the transcriptome of residual AML cells that were resistant to cytarabine treatment *in vivo* was highly enriched in genes involved in inflammatory and immune response, including the NF-kB network (71, 90). This gene signature of *in vivo* chemoresistance also displayed significant interactions with the dexamethasone gene signature for *FCGR1A*, *IL6ST*, *BIRC3*, *HGF*, *IL2RA*, *HDC*, *RHAG*, *STAT4*, *CALCRL*, *CD200*, and *CSF1* genes. Similarly, examination of a publicly available transcriptomic data set established from AML patients in first relapse and data mining algorithm revealed that the dexamethasone signature was also enriched within AML cells collected at relapse (71, 91). Moreover, in PDX models the dexamethasone-cytarabine combination induced a stronger therapeutic response compared to cytarabine alone. Overall, these data strongly suggest that the impact of dexamethasone with intensive chemotherapy that is observed in clinical practice could result from the targeting of inflammatory chemoresistant AML cells.

### The Action of Glucocorticoids on Leukemic Stem Cells

Leukemic long-term culture initiating cells (L-LTC-IC) are a reliable functional readout to monitor the activity of leukemia-initiating/stem cells (LICs), an AML subpopulation thought to be at the origin of relapse (89, 92). Using an optimized niche-like co-culture system capable of maintaining LICs *ex vivo*, dexamethasone reduced L-LTC-IC frequency and induced cellular differentiation (71). Furthermore, another recent study that combined the computational analysis of leukemic stem cell gene expression signatures with *in vitro* drug screening identified glucocorticoids as potent drugs that specifically target leukemic stem cells. In fact, glucocorticoids eliminated leukemic stem cell through differentiation induction, whereas they had no anti-leukemic activity against leukemic bulk (93). As described above, dexamethasone displays both cytoplasmic and nuclear activities that interfere with signal transducers or transcription factors such as PI3-kinase/Akt, activating protein-1 (AP-1), and NF-κB, which are all involved in leukemic stem-cell biology (73, 94, 95). It has been demonstrated that inflammatory cytokines can induce both NF-kB and AP-1 to support leukemic stem-cell survival in a synergistic manner (44). Therefore, by suppressing cytokine release and targeting specific intracellular pathways, dexamethasone may interfere with leukemic stem cell behavior and make them more susceptible to chemotherapy-induced cell death. Of course, the mechanisms of action underpinning dexamethasone activity in AML are likely to be multiple as leukemic stem cells are subject to different levels of regulation that are either cell autonomous or driven by interactions with the microenvironment (41, 96).

### The Action of Glucocorticoids in AML Subgroups

#### RUNX1-RUNX1T1

AML with t(8;21) translocation is induced by the oncogenic activity of the *RUNX1-RUNX1T1* fusion gene generated by chromosomal translocation and represents approximatively 10% of AML. The RUNX1-RUNX1T1 oncoprotein acts as a dominant negative regulator of RUNX1 transcriptional activity thereby repressing the expression of RUNX1-dependant genes involved in myeloid differentiation (97, 98). RUNX1-RUNX1T1 also promotes self-renewal of leukemic stem cells (99). In a chemogenomic screening of a small molecule library, two classes of compounds (glucocorticoids and dihydrofolate reductase inhibitors) were found to abrogate the *RUNX1-RUNX1T1* gene expression signature (100). Methylprednisolone and dexamethasone used at nanomolar concentrations induced cell differentiation and apoptosis in the Kasumi-1 cell line and the U937 cell line engineered to express *RUNX1-RUNX1T1* but not in other AML cell lines that do not express the oncoprotein. Glucocorticoid treatment was associated with a significant reduction in RUNX1-RUNX1T1 protein expression which was reverted by proteasome inhibitors, suggesting that glucocorticoids promote proteasome-mediated RUNX1-RUNX1T1 protein degradation. Of note, methylprednisolone treatment was associated with a down regulation of BCL-2 expression in this study and other investigators also found that dexamethasone down regulated BCL-X_L_ in the U937 cell line, which indicates that glucocorticoids may alter key anti-apoptotic molecules in myeloid cells (101). A more recent study also showed that glucocorticoid drugs can mimic *RUNX1-RUNX1T1* knock-down and induce both the inhibition of *RUNX1-RUNX1T1*-mediated gene expression and the stimulation of transcriptional activity of wild type *RUNX1* allele. Dexamethasone inhibited self-renewal of LICs and induced significant differentiation and apoptosis of RUNX1-RUNX1T1 AML cells (102). Moreover, dexamethasone used as a single agent inhibited tumor growth and prolonged the survival of mice engrafted with the Kasumi-1 cell line. Investigators proposed a mechanism of action by which glucocorticoids increased the amount of ligand-bound glucocorticoid receptors in the nucleus as well as their binding to DNA and *RUNX1*-target gene promoters. Interaction between glucocorticoid receptors and RUNX1 (but not RUNX1-RUNX1T1) increased RUNX1 binding to its target genes and decreased RUNX1-RUNX1T1 binding. The unbound RUNX1-RUNX1T1 might be subjected to proteasome-mediated degradation, resulting in a reduced level of oncoproteins in the nucleus. This leads to a significant change in gene expression with RUNX1 dominance over RUNX1-RUNX1T1 promoting RUNX1-mediated hematopoietic differentiation and inhibition of RUNX1-RUNX1T1-mediated stem cell maintenance. In addition, a combination of dexamethasone with cytarabine or doxorubicin was synergistic in this AML cell line, which suggests that combining dexamethasone with “3+7” induction and then with high-dose cytarabine during the consolidation phase could be a treatment option to be explored in patients with t(8;21)/RUNX1-RUNX1T1 AML (100, 102). It is also noteworthy that dexamethasone activity was stronger than prednisolone or hydrocortisone activity (102, 103).

#### *RUNX1* Mutations

AML with mutated *RUNX1* (*RUNX1*^mut^ AML) is a provisional entity that accounts for 10% of the newly diagnosed patients and is associated with a poor prognosis (104–107). In fact, *RUNX1*^mut^ AML is included in the high-risk group of the ELN 2017 classification. *RUNX1* mutations are frequently encountered in AML with minimal differentiation (AML-M0), normal karyotype or noncomplex karyotype with frequent association with trisomy 13. *RUNX1* mutations are mutually exclusive of recurrent translocations in AML and co-occur with *ASXL1* mutations and other gene mutations including epigenetic modifiers (*IDH2*, *KMT2A*, *EZH2*), spliceosome complex (*SRSF2*, *SF3B1*) and *STAG2*, *PHF6*, and *BCOR* (107). The RUNX1 mutation–associated gene and microRNA expression signatures revealed that the most strongly upregulated genes were related to hematopoietic stem/progenitor cells and/or B-cell progenitors, whereas genes normally expressed in myeloid-committed cells including CEBPA were among the most downregulated (108, 109). A recent study demonstrated that the *RUNX1* allele dosage may determine the *RUNX1* mutation-associated gene expression signature and identified a distinct *RUNX1*^mut^ AML subgroup with significant association with FAB M0 morphology, trisomy 13, and *ASXL1* mutations, whereas a chemogenomic approach revealed that AML samples bearing inactivating mutations of *RUNX1* were particularly sensitive to nanomolar concentrations of glucocorticoids (110). AML samples with dominant negative or *RUNX1^-/-^* mutations (lacking the wild type allele) were much more responsive to glucocorticoids than AML sample wild type RUNX1 allele or with missense *RUNX1* mutations, which are known to have no impact on RUNX1 function. Furthermore, down-regulation of *RUNX1* expression was associated with acquired dexamethasone sensitivity. Glucocorticoid sensitivity was reverted by the GR antagonist RU486 or *NR3C1* knock-down, which confirms that glucocorticoid activity in AML cells is dependent on their interaction with the GR. It is important to note that *RUNX1*^mut^ AML samples expressed increased levels of NR3C1 compared to most AML subgroups. Another study showed a similar acquisition of glucocorticoid sensitivity in blastic-phase CML cells with *RUNX1* mutations (111). However, at present there is no clinical data showing that glucocorticoids have an impact on *RUNX1*^mut^ AML.

#### *NPM1* Mutations

Mutations in the nucleophosmin (NPM1) gene are among the most frequent molecular alterations in AML (~35%) and represent a distinct entity in AML according to the 2016 WHO classification (112, 113). When the karyotype is normal and *FLT3-ITD* mutation is not associated or weakly expressed, *NPM1* mutation indicates a favorable outcome in AML according to the ELN 2017 prognosis classification even though the co-mutational environment may further influence this prognostic impact (6, 114). Unmutated NPM1 protein is mainly located in the nucleolus and plays a key role in the regulation of ribosome biogenesis, nucleolar function, protein synthesis, and tumor suppression by activating the TP53 pathway. Mutated NPM1 loses its predominant nucleolar location and accumulates in the cytoplasm thereby inducing leukemogenesis through *HOX* gene activation (115).

OCI-AML3, a *NPM1* mutated cell line, demonstrated *in vitro* and *in vivo* sensitivity to dexamethasone compared to wild type cell lines and primary AML samples with *NPM1* mutation were also more sensitive to dexamethasone-induced apoptosis than wild type samples (71, 110). Although, the mechanisms of action that sustain this particular sensitivity have not been described, *in silico* exploratory analyses showed that the *NPM1* mutation gene signature was highly enriched in genes responsive to dexamethasone, including upregulated genes such as *GGT1*, *CD86*, *NAMPT*, *ETS2*, *NFKBIA*, *PLA2G4A*, *IL1B*, *CD163*, *FPR1*, *HIST1H1C*, *CCL1*, *CXCL2*, *PTX3*, *TNF*, *RHAG*, *CCL20*, *DEFB1*, *CD300C*, *HOXB5*, and *IL6* (71).

#### *FLT3* Mutations

Mutations in the *FLT3* gene are also frequent mutations in AML that occur in up to 30% of patients (114). Two distinct *FLT3* mutations that induce constitutive ligand-independent activation of kinase are described: internal tandem duplications (ITD) in the juxtamembrane domain and point mutations in the tyrosine kinase domain (TKD). *FLT3* mutations are associated with an aggressive disease course especially *FLT3*-ITD which predicts early relapse and a poor prognosis. Through clonal selection under chemotherapy, a higher mutant allelic burden is frequently observed at relapse, which indicates that AML cells have become more addicted to FLT3 signaling. In the preclinical setting, *FLT3*-mutant allelic burden and clinical status (diagnosis versus relapse samples) are predictive of a response to FLT3 inhibitors in AML (116). Furthermore, *FLT3*-ITD is also an independent factor of a poor prognosis in R/R AML (117, 118). Recently, two class I FLT3 inhibitors that target both *FLT3*-ITD and *FLT3*-TKD mutations were approved for the treatment of AML with *FLT3* mutation. Midostaurin was approved in combination with intensive chemotherapy as a first line treatment whereas gilteritinib was approved as a single agent treatment in relapsed or refractory patients (119, 120).

A very recent study aimed at describing the mechanisms of early acquired resistance to FLT3 inhibitors showed that AML cells with *FLT3*-ITD mutations that persist after 48 h of FLT3 inhibitor drug exposure (drug-tolerant persisters) up-regulated both the inflammatory response gene and GR expression. This resulted in synergistic activity of the combination of FLT3 inhibitors and glucocorticoids through apoptosis induction both *in vitro* and in xenotransplantation mice models (121). This very rapid and transient resistance mechanism was specific to FLT3 mutated cells and not due to the selection of resistance-conferring mutation or the reactivation of FLT3 signaling. Rather, it was dependent on the up-regulation of GC receptor expression upon FLT3i exposure and the increased BIM to MCL1 ratio upon combination treatment. To be more specific, BIM expression was strongly up-regulated by glucocorticoids whereas MCL1 was down-regulated by FLT3i through GSK-mediated activation of the proteasome pathway and MCL1 protein degradation. This preclinical study should stimulate clinical trials in this setting since the complete response rate with the recently approved gilteritinib is < 50%.

#### Spliceosome-Complex Mutations

Genes of the spliceosome-complex including *SRSF2*, *SF3B1*, *U2AF1*, and *ZRSR2* are frequently mutated in myeloid malignancies such as AML, myelodysplastic syndromes (MDS) or myeloproliferative disorders (122). In AML, they are particularly found in patients with a history of MDS or chronic myelomonocytic leukemia (secondary AML) (123). These mutations induce neomorphic or gain-of-function splicing phenotypes and alter the splicing of many genes (124). Recently, it was shown that an increase in inflammatory cytokine production including IL-6, IL-8, TNF-α and NF-kB activation is found in leukemic cells harboring spliceosome mutations (125–127). Moreover, *SRSF2*-mutated AML samples also expressed an increase in the level of *NR3C1* and were responsive to glucocorticoid treatment (110). However, the activity of glucocorticoids on other spliceosome-complex mutations has not been assessed to date.

## Perspectives: Will There Be a Place for Dexamethasone in AML Treatment?

**Figure 3:**
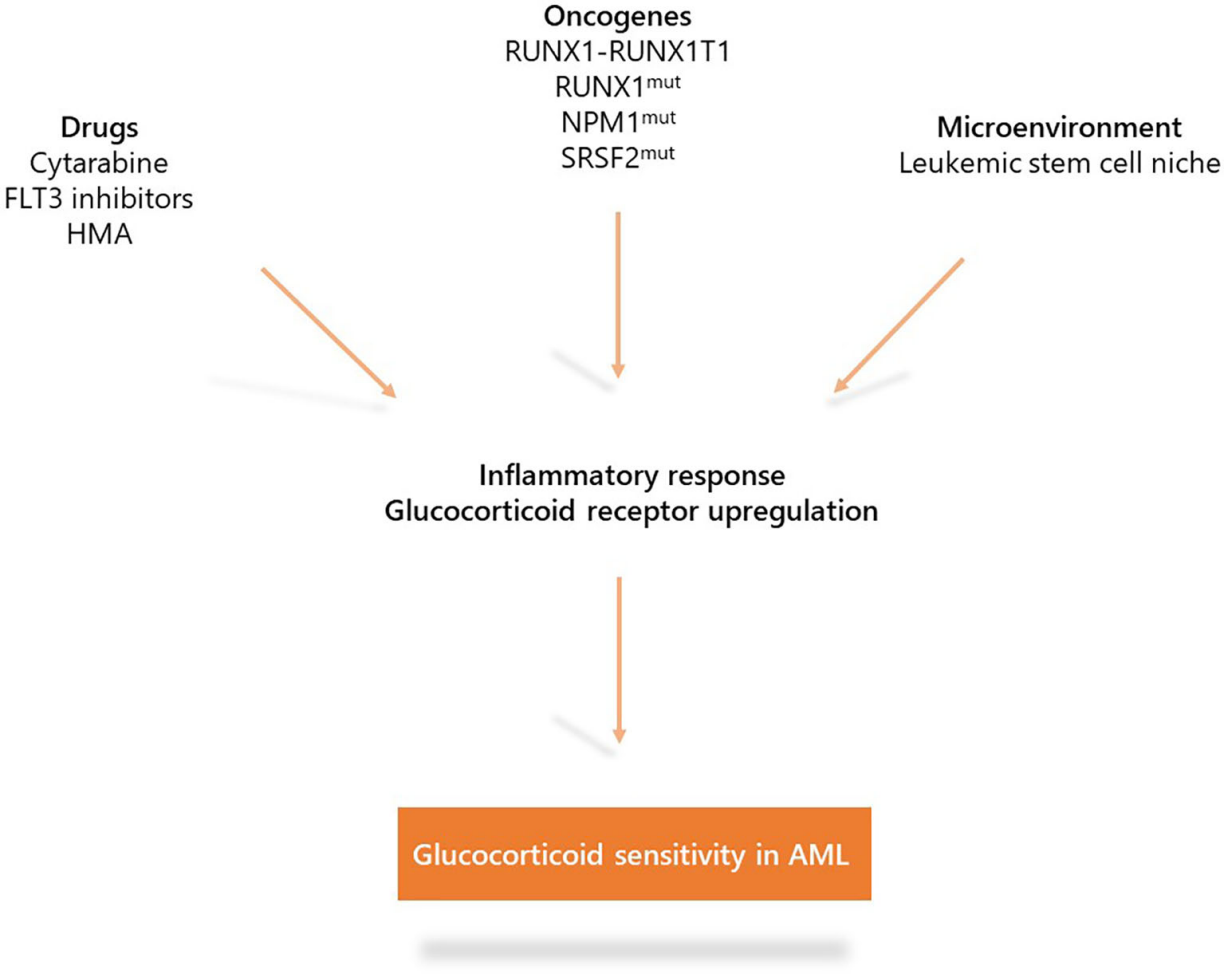
Mechanisms that induce glucocorticoid sensitivity in acute myeloid leukemia (AML). Specific oncogenic alterations (*RUNX1-RUNX1T1*, *RUNX1*, *NPM1*, or *SRSF2* mutations as well as NF-kB/AP-1 activation in LSCs) or therapeutic stress (cytarabine, FLT3 inhibitors) are associated with up-regulation of the inflammatory gene response and expression of the glucocorticoid receptor which induce sensitization to glucocorticoid treatment.

**Figure 4:**
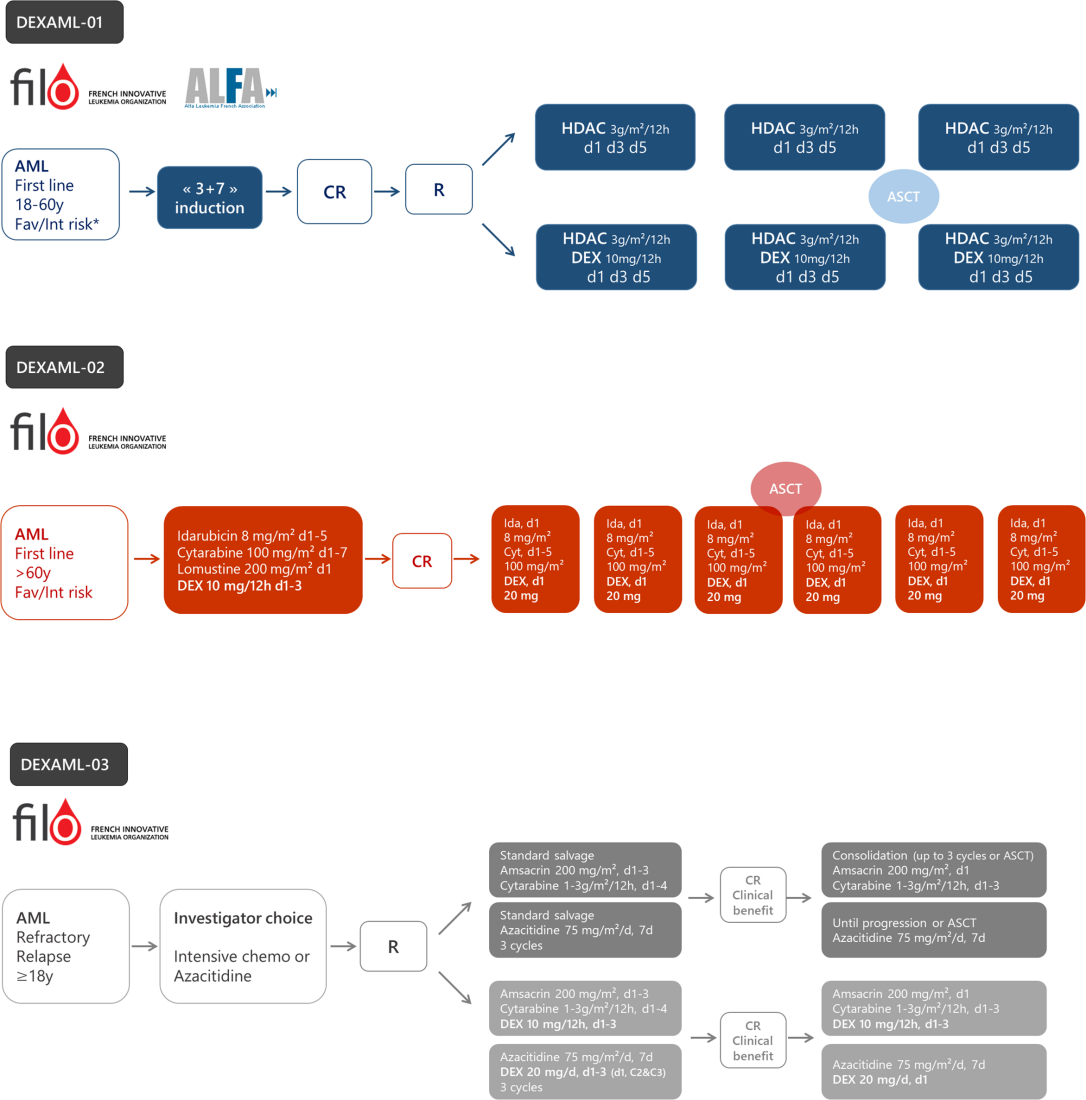
The design of ongoing prospective trials to evaluate dexamethasone in acute myeloid leukemia (AML). DEXAML-01: patients (18–60 years) with favorable (fav) or intermediate (int) risk in first complete response after induction chemotherapy. DEXAML-02: patients > 60 with favorable or intermediate risk in first line treatment. DEXAML-03: patients > 18 years with refractory or relapsed AML. CR, complete response; R, randomization; HDAC, high dose cytarabine (3 g/m²/12 h on day 1, 3, and 5); DEX, dexamethasone.

These recent studies indicate the role of inflammation in AML biology. Under specific oncogenic (*RUNX1*, *NPM1*, or *SRSF2* mutations and NF-kB activation in LSCs) or therapeutic (cytarabine and FLT3 inhibitors) stress, AML displays up-regulation of the inflammation gene response and GR expression that induces sensitization to glucocorticoid therapeutic action partially through the modulation of key apoptotic protein (**Figure 3**). Whether other oncogenes involved in AML such as *IDH1/2*, *TP53*, *KMT2A*, or *EVI-1* or other treatments including hypomethylating agents or BCL2 inhibitors may also induce inflammatory phenotypes remains to be determined. Therefore, although the glucocorticoid unresponsiveness of AML bulk is an established preclinical and clinical fact which to date has precluded clinical investigations on AML unlike ALL, some AML molecular subgroups or cell subpopulations could benefit from therapeutic intervention with glucocorticoids. Therefore, the French cooperative FILO study group designed three prospective clinical trials in the field (**Figure 4**).

### Dexamethasone in Hyperleukocytic AML (DEXAML-00)

AML patients with a high white blood-cell count are difficult to manage, require immediate chemotherapy induction, and therefore are often excluded from prospective trials. In the retrospective DEXAML-00 study in patients with hyperleukocytic AML, the impact of dexamethasone on all survival endpoints and the incidence of relapse, was adjusted according to several clinical and biological factors to limit the potential biases inherent in non-randomized studies (71). As indicated above, dexamethasone may also be of benefit in critically-ill AML patients (66, 70, 128, 129). Therefore, many French centers, including ours, are accustomed to treating all patients with a white blood cell count >50 G/L with a short course of dexamethasone in combination with chemotherapy. Previous studies on dexamethasone pharmacokinetics have shown that plasma levels achieved in dosed patients with this schedule are likely to reach therapeutic concentrations used in preclinical AML models (130, 131). Moreover, relevant logical biological bases strengthened these retrospective clinical findings and paved the way to further explore dexamethasone action in other situations besides hyperleukocytic AML.

### Dexamethasone With High-Dose Cytarabine as Post-Remission Therapy in Younger AML Patients (DEXAML-01)

Based on preclinical evidence showing increased sensitivity to glucocorticoids in cytarabine-resistant cells and in LSC’s assumed to be at the origin of relapse, dexamethasone is currently being tested in a phase II-III randomized trial within the French ALFA/FILO backbone intergroup-1 trial (BIG-1, NCT02416388). Younger patients (18–60 years) with favorable or intermediate risk AML, in their first complete remission following standard induction chemotherapy, receive either high-dose cytarabine alone (standard of care) or high-dose cytarabine plus dexamethasone (**Figure 4**). The primary objective is to improve leukemia-free survival with the addition of dexamethasone. To date, 178 patients have been randomized and recruitment should be completed during the first half of 2021 for a total of 220 patients. The final analysis will be performed 18 months after the last inclusion.

### Dexamethasone With Intensive Chemotherapy as First-Line Therapy in Older AML Patients (DEXAML-02)

The FILO study group induction chemotherapy for newly diagnosed AML patients older than 60 is a 3-drug regimen including idarubicin, cytarabine, and lomustine (ICL) (132). The DEXAML-02 trial is a prospective, single arm, phase 2 trial that evaluates the addition of dexamethasone during both induction and consolidation (NCT03609060). The primary objective is to determine whether adding dexamethasone to ICL induction and IC post-remission therapy results in significant improvement of event-free survival compared with a historical cohort of the FILO LAM-SA 2007 trial (132). Of note, midostaurin is added in FLT3 mutated patients. FILO was not able to conduct a randomized trial versus placebo since the proposal was not considered a high enough priority by the national clinical research funding agency. Recruitment of the 120 patients ended in early 2020. Ancillary studies on leukemic stem cells, immune subpopulations and cytokine production subsequent to treatment are planned. The final analysis will be performed in 2022.

### Dexamethasone With Intensive Salvage Chemotherapy or Azacitidine in Relapsed/Refractory AML (DEXAML-03)

AML patients who fail to achieve a complete response with standard induction therapy (refractory AML) or who relapse after achieving remission have a very poor prognosis (133). Treatment of relapsed or refractory (R/R) AML in patients who are candidates for intensive salvage chemotherapy consists of reducing the leukemia burden in order to achieve remission before allogeneic stem-cell transplantation, which is currently the treatment with the highest probability for cure. The prognosis is even more dismal in patients deemed unfit for intensive chemotherapy. Non-intensive approaches including hypomethylating agents are generally proposed in this context (134).

As indicated above, AML samples collected at relapse are much more sensitive to glucocorticoids than diagnosis samples (82, 84). Moreover, preclinical studies have shown that the gene signature of cells resistant to hypomethylating agents is also enriched with dexamethasone response genes (135). In addition, in ALL cell lines, azacitidine has been shown to restore GR expression and sensitivity to dexamethasone (136). Therefore, a randomized phase 3 clinical trial was designed to assess the impact of dexamethasone added to either intensive chemotherapy or azacitidine, according to the investigator’s choice, in adult patients with R/R AML (NCT03765541). The primary objective is to evaluate whether the addition of dexamethasone to salvage therapy significantly improves overall survival. This study is ongoing.

## Conclusion

Repositioning approved drugs for other indications is an interesting subject for clinical trials by cooperating study groups (137). Even though glucocorticoids have received little attention in AML, the evidence in this review provides a basis for clinical assessment of whether glucocorticoids such as dexamethasone can one day be included in the AML treatment armamentarium. A panel of 79 myeloid genes will be screened by next-generation sequencing in the DEXAML studies to explore the clinical benefit of dexamethasone in specific subgroups as suggested by several preclinical studies. If the results of these clinical trials are positive, they could be easily and widely incorporated into routine practice since hematologists have long experience with this drug in lymphoid malignancies.

## Author Contributions

The author confirms being the sole contributor of this work and has approved it for publication.

## Funding

Funding from the Fondation Toulouse Cancer Santé, Force Hémato (AO 2017), the French Ministry of Health (PHRC interrégional 2017) and the Ligue Régionale Contre le Cancer (AO 2018).

## Conflict of Interest

The author declares that the research was conducted in the absence of any commercial or financial relationships that could be construed as a potential conflict of interest.
